# Enrichment of high-grade tumors in breast cancer gene expression studies

**DOI:** 10.1007/s10549-017-4622-9

**Published:** 2017-12-18

**Authors:** M. van Seijen, A. L. Mooyaart, L. Mulder, M. Hoogstraat, C. A. Drukker, C. E. Loo, B. Pouw, G. S. Sonke, J. Wesseling, E. H. Lips

**Affiliations:** 1 Department of Molecular Pathology, The Netherlands Cancer Institute, Plesmanlaan 121, 1066 CX Amsterdam, The Netherlands; 2 Department of Pathology, VU University Medical Centre, De Boelelaan 1117, 1081 HV Amsterdam, The Netherlands; 3 Department of Pathology, Erasmus University Medical Center, Wytemaweg 80, 3015 CN Rotterdam, The Netherlands; 4 Department of Pathology, The Netherlands Cancer Institute, Plesmanlaan 121, 1066 CX Amsterdam, The Netherlands; 5 Department of Molecular Carcinogenesis, The Netherlands Cancer Institute, Plesmanlaan 121, 1066 CX Amsterdam, The Netherlands; 6 Department of Surgery, The Netherlands Cancer Institute, Plesmanlaan 121, 1066 CX Amsterdam, The Netherlands; 7 Department of Radiology, The Netherlands Cancer Institute, Plesmanlaan 121, 1066 CX Amsterdam, The Netherlands; 8 Department of Medical Oncology, The Netherlands Cancer Institute, Plesmanlaan 121, 1066 CX Amsterdam, The Netherlands

**Keywords:** Breast cancer, Gene expression, Profiling, Selection bias, Tumor percentage

## Abstract

**Purpose:**

Gene expression (GE) profiling for breast cancer classification and prognostication has become increasingly used in clinical diagnostics. GE profiling requires a reasonable tumor cell percentage and high-quality RNA. As a consequence, a certain amount of samples drop out. If tumor characteristics are different between samples included and excluded from GE profiling, this can lead to bias. Therefore, we assessed whether patient and tumor characteristics differ between tumors suitable or unsuitable for generating GE profiles in breast cancer.

**Methods:**

In a consecutive cohort of 738 breast cancer patients who received neoadjuvant chemotherapy at the Netherlands Cancer Institute, GE profiling was performed. We compared tumor characteristics and treatment outcome between patients included and excluded from GE profiling. Results were validated in an independent cohort of 812 patients treated with primary surgery.

**Results:**

GE analysis could be performed in 53% of the samples. Patients with tumor GE profiles more often had high-grade tumors [odds ratio 2.57 (95%CI 1.77–3.72), *p* < 0.001] and were more often lymph node positive [odds ratio 1.50 (95%CI 1.03–2.19), *p* = 0.035] compared to the group for which GE profiling was not possible. In the validation cohort, tumors suitable for gene expression analysis were more often high grade.

**Conclusions:**

In our gene expression studies, tumors suitable for GE profiling had more often an unfavorable prognostic profile. Due to selection of samples with a high tumor percentage, we automatically select for tumors with specific features, i.e., tumors with a higher grade and lymph node involvement. It is important to be aware of this phenomenon when performing gene expression analysis in a research or clinical context.

**Electronic supplementary material:**

The online version of this article (10.1007/s10549-017-4622-9) contains supplementary material, which is available to authorized users.

## Introduction

DNA microarray analyses (e.g., tiling arrays, mRNA arrays, and direct sequencing of complementary DNA) have significantly advanced our understanding of breast cancer. They showed that breast cancer is not a single disease with variable morphologic features, but a group of molecular distinct neoplasms [1]. Furthermore, in certain clinical settings it can help to determine whether or not adjuvant chemotherapy is justified [2].

Several assays, resulting in risk scores, have been developed and are partially commercially and partially clinically available. The 21-gene Recurrence Score (OncotypeDx assay, Genome Health inc, Redwood city, CA) [3], the Amsterdam 70-gene profile, commercially known as the Mammaprint (Agendia, Huntington Beach, CA) assay [4] and the Risk of Recurrence (ROR) score, derived from Predictor Analysis of Microarray 50 (PAM50) [5], are mostly used. Reliable results of the assays require a good quality tumor sample with high cellularity. To illustrate this, Elloumi et al. [6] revealed a systematic bias when too much normal tissue was present in a tumor sample. However, tumor percentage is also dependent on tumor morphology. For example, a tumor with solid growth more easily reaches a high tumor percentage than a tumor with glandular or lobular growth (a feature important for grade). Furthermore, the presence of sclerosis as well as stromal and inflammatory cells can reduce the tumor cell percentage substantially. Another feature that influences the ability to perform gene expression analysis is the RNA quality. Pre-analytical factors such as time to fixation, fixation duration, and storage temperature have an impact on the RNA quality [7].

Summarizing the above, a high tumor percentage and good quality RNA are prerequisites for successful gene expression analysis. These requirements lead to the dropout of samples not fulfilling these criteria. As a higher histological grade is associated with a higher tumor cellularity, gene expression analysis might be more successful in high-grade tumors. To assess if indeed clinico-pathological variables are associated with successful gene expression analysis, we compared clinical and tumor characteristics of tumors suitable and unsuitable for gene expression analysis in two large (neo) adjuvant-treated patient cohorts.

## Materials and methods

### Patient selection

Tissue samples of patients treated in the neoadjuvant setting for breast cancer were collected at the Netherlands Cancer Institute (NKI) between 2004 and 2012. For participation in the neoadjuvant program, the tumor diameter should exceed 3 cm or axillary lymph node metastasis should be proven by fine-needle aspiration. Part of these patients participated in two ongoing clinical trials of which details have been described previously [8]. Both studies were approved by the ethical committee and informed consent for gene expression analysis was obtained for all included patients. At least two tumor biopsies were taken under ultrasound guidance, using a 14G core needle to assure sufficient tissue for both adequate diagnostics as well as for research purposes. To facilitate such analyses, at least one biopsy was snap frozen in liquid nitrogen and stored at − 80 degrees.

### Pathology

Paraffin-embedded sections were all stained by a hematoxylin and eosin (H&E) stain and reviewed by a consultant breast pathologist (JW) for immunohistochemically assessment and histological classification (including subtype and grade) on biopsy material (of which the details are described previously) [9]. In brief, samples were scored as positive for oestrogen receptor (ER) and progesterone receptor (PR) if at least 10% of the tumor cells showed nuclear staining. *HER2* (or *ERBB2*) was scored as positive when there was strong membranous staining in more than 30% of the tumor cells (3+) by immunohistochemistry or if chromosome in situ hybridization (CISH) revealed amplification. Percentage nuclear staining of tumor cells in Ki67 (MIB1) was scored as a marker for proliferation. Chemotherapy response was determined by pathological examination of resection specimens. Pathological complete remission (pCR) was defined as the absence of invasive tumor in both the breast and axillary lymph nodes after neoadjuvant chemotherapy.

### Imaging data

For a subset of the patients, detailed imaging data were available. A dedicated breast radiologist (CL) assessed according to BIRADS lexicon [10] whether pre-treatment MRIs showed the tumor to be either mass-like, or non-mass like. For analysis purposes, these two categories were used. Metabolic activity was assessed using baseline 18F-fluorodeoxyglucose (FDG) positron emission tomography combined with computed tomography (PET/CT) scans. FDG uptake was quantified using maximum standardized uptake values (SUVmax) measured with Osirix DICOM viewer (Pixmeo SARL, Geneva, Swiss).

### Gene expression assay and tumor percentage

mRNA was isolated from the frozen material as described previously [9]. Briefly, a 5-micrometer section of the biopsy was H&E stained. A pathologist evaluated if the overall tissue quality of the frozen biopsy was sufficient for further analysis (i.e., samples dropped out if the biopsy was too small, too fatty or in the absence of invasive tumor). The pathologist also estimated the tumor percentage and only the samples with a tumor percentage ≥ 50% were selected for microarray analysis. Gene expression analysis was only performed if the RNA integrity number ≥ 6.5 and the quantity ≥ 3 µg. Samples obtained between 2004 and 2010 were analyzed using Illumina microarray analysis (WG6 v3 microarray chips); RNAseq was performed on the samples from 2011 to 2012.

### Validation cohort

As a validation, an independent cohort obtained from the microarRAy PrognoSTics in Breast cancER (RASTER) study was used. Study design is described before [11]. In short, 812 women were enrolled in 16 hospitals in The Netherlands after having given informed consent. Patients received surgery (mastectomy or breast conserving surgery) as primary treatment. Within 1 h after surgery, a tumor sample was procured at the pathology department of the participating hospitals and sent to Netherlands Cancer institute by mail. After samples were received at the Netherlands Cancer Institute they were snap frozen at − 70 degrees. Pathologists analyzed paraffin-embedded tumor samples of the validation dataset at the pathology department of the participating hospitals. Histological tumor grade according Elston and Ellis, ER status, PR status, and ERBB2 status were established by each hospital according to locally used methods [11]. Frozen sections of the tumor samples of the validation set were obtained and stained with H&E stain, and subsequently analyzed by an experienced breast pathologist. Eligible samples had to contain ≥ 50% tumor cells. Agendia Laboratories performed the microarray analysis using the Mammaprint (Agilent microarray, Santa Clara USA) [11].

### Data analyses and statistics

The variables age, histologic subtype of tumor, grade, T-stage, N-stage, and response (pathological complete remission (pCR) of breast and axilla) were compared between samples suitable or unsuitable for gene expression analysis. The *χ* ^2^ (Spearman) was used to compare dichotomized variables. We also assessed differences in clinical characteristics for each exclusion criteria as described above, i.e., tissue quality of the frozen biopsy, tumor cell percentage, and RNA quantity as well as quality. Multivariate logistic regression analysis was performed to assess the independent association of various clinical variables with the ability to perform GE analysis. Recurrence-free survival was assessed with Kaplan–Meier plots and the log rank test. A cox proportional hazard model was built to assess if the ability to perform GE analysis was independently associated with recurrence-free survival. The SPSS Package 23.0 was used for statistical analyses and *p* values (two-sided) < 0.05 were considered statistically significant. This study was designed according to the Reporting recommendations for tumor MARKER prognostic studies (REMARK) guidelines [12].

For the validation dataset, the variables age, histologic type of tumor, subtype, histological grade, and T-stage were compared between tumor samples suitable or ineligible for gene expression analysis. Statistical analysis was performed as described above.

## Results

### Patient selection in cohort

**Fig. 1:**
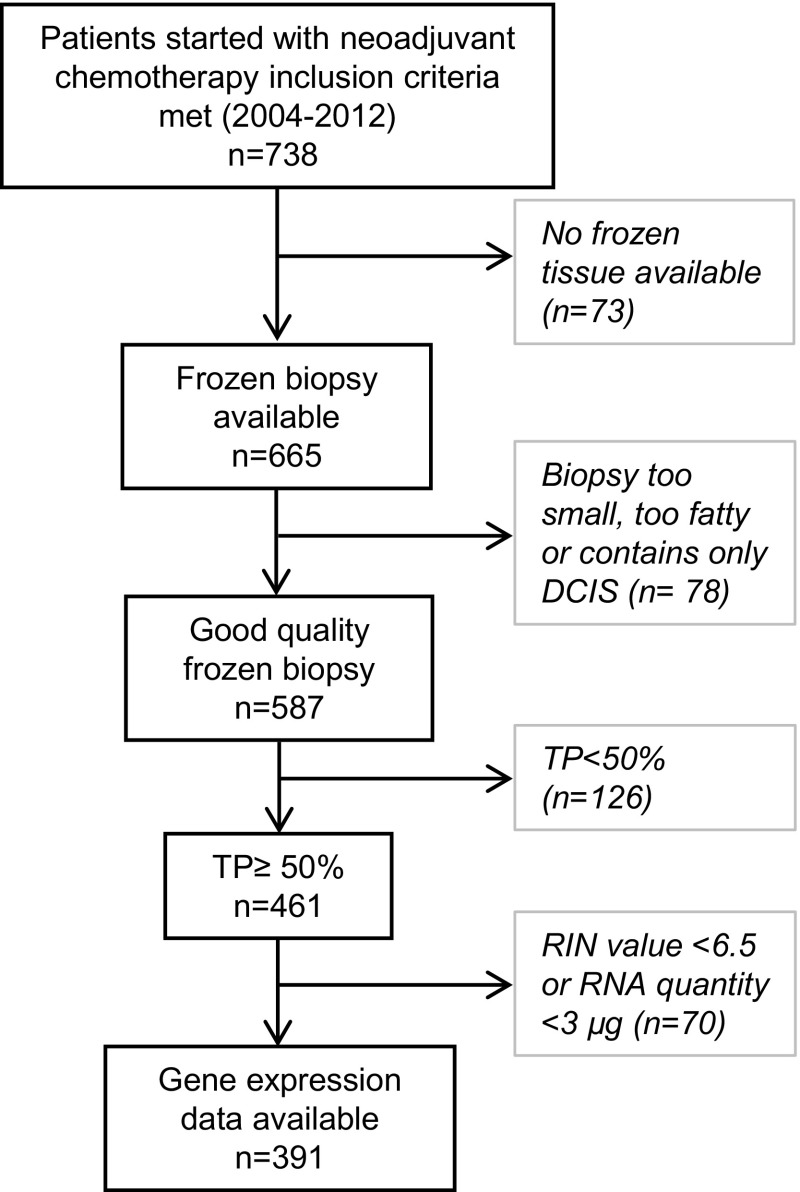
Flow diagram of included patients. Flow diagram of included patients and in- and exclusion steps of the fresh frozen biopsies for gene expression studies. *TP* denotes tumor percentage, *DCIS* ductal carcinoma in situ, *RIN* RNA integrity number

A total of 738 breast cancer patients were treated in the NKI with neoadjuvant chemotherapy between 2004 and 2012. From 665 patients, a frozen biopsy was available. Seventy-seven tissue samples were not processed because the biopsies were too small or too fatty, or did not contain invasive tumor. Of the remaining 587 tissue specimens, 461 had a tumor percentage of more than 50%; 391 of these samples met the criteria for sufficient RNA quality and quantity, allowing gene expression analysis (53% of the total cohort). Figure 1 shows the sample selection flow diagram.

### Association with clinical characteristics

Comparisons of baseline characteristics between the tumors for which a gene expression (GE) profile could be generated (GE+ ; *n* = 391) and the tumors for which this was not possible (GE− ; *n* = 347) are shown in Table 1. GE + tumors were more often high grade and had a higher SUV max value than GE− tumors. When we stratify for subtype, the effect of grade is still significant in the ER+HER2− and in the triple-negative subgroup (see Supplementary, Table 1). In the ER+HER2− subgroup, these samples also have a higher SUV max value. Multivariate analysis shows that a high tumor grade and positive lymph node status are independently associated with GE + tumors (Table 2).

**Table 1:** Characteristics of patients with gene expression profiles versus patients without gene expression profiles

		No GE data available (*n* = 347)	GE data available (*n* = 391)	*p* value
Age	< 50	189 (55%)	237 (61%)	0.13
	> = 50	153 (45%)	153 (39%)	
	Unknown	5	1	
Histology	IDC	244 (87%)	291 (90%)	0.25
	ILC	38 (13%)	34 (10%)	
	Unknown	65	66	
Subtype	ER+, Her2−	182 (53%)	182 (47%)	0.25
	Her2+	73 (21%)	96 (25%)	
	Tripleneg	91 (26%)	113 (29%)	
	Unknown	1	0	
ER	ER neg	122 (35%)	149 (38%)	0.44
	ER pos	223 (65%)	242 (62%)	
	Unknown	2	0	
HER2	Her2 neg	272 (79%)	293 (75%)	0.26
	Her2 pos	73 (21%)	96 (25%)	
	Unknown	2	2	
Grade	Grade 1 & 2	184 (70%)	158 (48%)	**<** **0.01**
	Grade 3	80 (30%)	170 (52%)	
	Unknown	83	63	
T-stage	T1/T2	232 (68%)	270(70%)	0.65
	T3/T4	109 (32%)	118 (30%)	
	Unknown	6	3	
N-stage	Neg	104 (30%)	94 (24%)	0.06
	Pos	239 (70%)	294 (76%)	
	Unknown	4	3	
Response (breast and lymph node)	No pCR	270 (79%)	293 (75%)	0.22
	pCR	72 (21%)	97 (25%)	
	Unknown	5	1	
Ki-67	> 15%	103 (49%)	98 (49%)	0.95
	≤ 15%	107 (51%)	103 (51%)	
	Unknown	137	190	
Mass (MRI)	Non-mass	47 (55%)	53 (55%)	0.94
	Mass	39 (45%)	43 (45%)	
	Unknown	261	295	
Maximal SUV-uptake mean (sd)		7.19 (4.55)	10.03 (6.79)	**0.03**
Unknown		259	294	

Due to rounding, some percentages do not count up to 100%. *GE* denoted gene expression, *IDC* invasive ductal carcinoma, *ILC* invasive lobular carcinoma, *ER* oestrogen receptor, *HER2* ERBB2, *SUV* standardized uptake value

Bold *p*-values denote *p*-values statitically significant at a 0.05 cut-off

**Table 2:** Multivariate analyses of patient characteristics with gene expression profiles versus patients without gene expression profiles

Variable		Frequency	Odds ratio	95% CI	*p* value
Age	< 50	339	1.00		
	> = 50	245	0.91	0.64–1.28	0.58
ER	ER neg	218	1.00		
	ER pos	366	1.19	0.81–1.74	0.38
HER2	Her2 neg	440	1.00		
	Her2 pos	144	1.20	0.81–1.80	0.36
Grade	Grade 1 & 2	336	1.00		
	Grade 3	248	2.56	1.77–3.71	**<** **0.01**
T-stage	T1/T2	408	1.00		
	T3/T4	176	0.94	0.65–1.35	0.73
N-stage	Neg	163	1.00		
	Pos	421	1.53	1.05–2.23	**0.03**

An odds ratio above one means that gene expression analysis is more likely in this patient group. *ER* denoted oestrogen receptor, *HER2* ERBB2

Bold *p*-values denote *p*-values statitically significant at a 0.05 cut-off

To investigate the influence of the various steps of sample selection for gene expression analysis in more detail, we compared the clinical variables between in- and excluded samples after each selection step (Table 3). Interestingly, in every selection step, we enrich for high-grade tumors: a high tumor grade is associated with larger biopsies, higher tumor percentage and high quality and quantity RNA. In addition, high quality and sufficient quantity of RNA is more often found in HER2+ tumors and node-positive tumors.

### Association with treatment response and survival

**Fig. 2:**
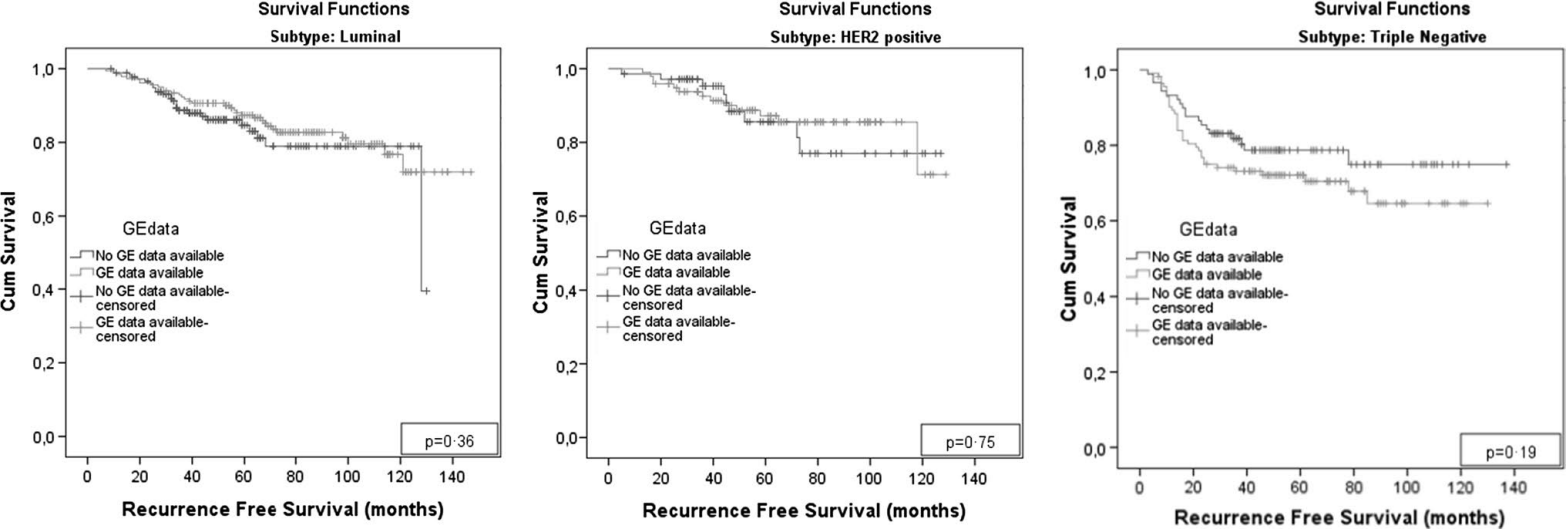
Kaplan–Meier curves of recurrence-free survival. Kaplan–Meier curves of recurrence-free survival to compare patients with and without gene expression profiles, stratified by subtype. GE denoted gene expression

Chemotherapy response was not significantly different between GE + and GE − tumors. However, we observed that tumors with high tissue quality of frozen biopsies more often achieved a pathological compete response (pCR) after treatment than samples with poor quality biopsies (*p* = 0.02; Table 3). We did not observe a significant difference in recurrence-free survival between samples included and excluded in gene expression analysis, although a trend was visible in triple-negative patients (Fig. 2). A cox proportional hazard model did not indicate GE+ as a variable associated with survival (Supplementary, Table 2).

**Table 3:** Characteristics of patients excluded versus remaining group. Due to rounding, some percentages do not count up to 100%

		Frozen tissue available			Quality of frozen biopsy			Tumor %			RNA quality and quantity		
		No	Yes	*p* value	Insufficient	Sufficient	*p* value	< 50%	> = 50%	*p* value	Insufficient	Sufficient	*p* value
		(*n* = 73)	(*n* = 665)		(*n* = 78)	(*n* = 587)		(*n* = 126)	(*n* = 461)		(*n* = 70)	(*n* = 391)	
Age	<50	44 (62%)	382 (58%)	0.50	34 (45%)	348 (59%)	**0.02**	77 (61%)	271 (59%)	0.66	34 (49%)	237 (61%)	0.06
	> = 50	27 (38%)	279 (42%)		41 (55%)	238 (41%)		49 (39%)	189 (41%)		36 (51%)	153 (39%)	
	Unknown	2	4		3	1		0	1		0	1	
Histology	IDC	58 (92%)	477 (88%)	0.31	49 (85%)	428 (88%)	0.43	88 (87%)	340 (88%)	0.74	49 (82%)	291 (90%)	0.08
	ILC	5 (8%)	67 (12%)		9 (16%)	58 (12%)		13 (13%)	45 (12%)		11 (18%)	34 (11%)	
	Unknown	10	121		20	101		25	76		10	66	
Subtype	ER+,Her2−	33 (45%)	331 (50%)	0.62	46 (60%)	285 (49%)	0.18	62 (49%)	223 (48%)	0.79	41 (59%)	182 (47%)	**0.04**
	Her2+	20 (28%)	150 (23%)		14 (18%)	136 (23%)		31 (24%)	104 (23%)		8 (11%)	96 (25%)	
	Triple negative	20 (28%)	183 (27%)		17 (22%)	167 (28%)		33 (26%)	134 (29%)		21 (30%)	113 (29%)	
	Unknown	0	1		1	0		0	0		0	0	
ER	ER neg	26 (36%)	245 (37%)	0.82	24 (32%)	221 (38%)	0.30	48 (38%)	173 (38%)	0.91	24 (34%)	149 (38%)	0.54
	ER pos	47 (64%)	418 (63%)		52 (68%)	366 (62%)		78 (62%)	288 (63%)		46 (66%)	242 (62%)	
	Unknown	2	2		2	0		0	0		0	0	
Her2	Her2 Neg	52 (72%)	513 (78%)	0.31	63 (82%)	450 (77%)	0.33	95 (75%)	355 (77%)	0.65	62 (89%)	293 (75%)	**0.02**
	Her2 pos	20 (28%)	149 (22%)		14 (18%)	135 (23%)		31 (25%)	104 (23%)		8 (11%)	96 (25%)	
	Unknown	1	3		1	2		0	2		0	2	
Grade	Grade 1 & 2	36 (72%)	306 (57%)	**0.03**	43 (72%)	263 (55%)	**0.01**	61 (66%)	202 (52%)	**0.02**	44 (72%)	158 (48%)	**<** **0.01**
	Grade 3	14 (28%)	236 (43%)		17 (28%)	219 (45%)		32 (34%)	187 (48%)		17 (28%)	170 (52%)	
	Unknown	23	123		18	105		33	72		9	63	
T-stage	T1/T2	43 (61%)	459 (70%)	0.11	58 (78%)	401 (69%)	0.09	84 (67%)	317 (69%)	0.59	47 (67%)	270 (70%)	0.68
	T3/T4	28 (39%)	199 (30%)		16 (22%)	183 (31%)		42 (33%)	141 (31%)		23 (33%)	118 (30%)	
	Unknown	2	7		4	3		0	3		0	3	
N-stage	Neg	21 (29%)	177 (27%)	0.68	21 (28%)	156 (27%)	0.81	35 (28%)	121 (26%)	0.76	27 (39%)	94 (24%)	**0.01**
	Pos	51 (71%)	482 (83%)		54 (72%)	428 (73%)		91 (72%)	337 (74%)		43 (61%)	294 (76%)	
	Unknown	1	6		3	3		0	3		0	3	
Response	No pCR	55 (76%)	508 (77%)	0.91	65 (88%)	443 (76%)	**0.02**	98 (78%)	345 (75%)	0.52	52 (74%)	293 (75%)	0.88
	pCR	17 (24%)	152 (23%)		9 (12%)	143 (24%)		28 (22%)	115 (25%)		18 (26%)	97 (25%)	
	Unknown	1	5		4	1		0	1		0	1	

*IDC* denoted invasive ductal carcinoma, *ILC* invasive lobular carcinoma, *ER* oestrogen receptor, *HER2* ERBB2, *pCR* pathological complete response

Bold *p*-values denote *p*-values statitically significant at a 0.05 cut-off

### Results in the validation dataset

The RASTER data were used to validate our observations. This set includes 812 breast cancer patients enrolled between 2004 and 2006 (see Methods). Because node positive patients were excluded in this study for clinical reasons and therefore a gene expression profile was not performed, we analyzed the samples of node-negative patients (*n* = 585, see Supplementary Fig. 1, for a flowchart). Therefore, we could not validate our association with nodal status in this set; however, most other clinical variables were available. Of these samples, 27% dropped out because of incorrect procedure or sample failure. Similar to the observations in the neoadjuvant cohort, gene expression profiling was more often possible in high-grade tumors (borderline significant; *p* = 0.05, Table 4).

**Table 4:** Patient characteristics in the validation dataset, split for gene expression status

		*n* = 585		
		No GE data available (*n* = 158)	GE data available (*n* = 427)	*p* value
Age	< 50 yr	86 (54%)	251 (59%)	0.34
	≥ 50 yr	72 (46%)	176 (41%)	
	Unknown	0	0	
Histology	IDC	125 (79%)	345 (81%)	0.11
	ILC	12 (7%)	47 (11%)	
	Rest	21 (13%)	35 (8%)	
	Unknown	0	0	
Subtype	Luminal	121 (77%)	312 (73%)	0.49
	Her2	19 (12%)	48 (11%)	
	Triple negative	18 (11%)	65 (15%)	
	Unknown	0	2	
ER	ER Neg	23 (15%)	85 (20%)	0.14
	ER Pos	135 (85%)	342 (80%)	
	Unknown	0	0	
Her2	Her2 Neg	119 (75%)	358 (88%)	0.55
	Her2 Pos	19 (14%)	48 (1%)	
	unknown	20	21	
Grade	Grade 1 & 2	120 (76%)	291 (68%)	**0.05**
	Grade 3	37 (24%)	136 (32%)	
	Unknown	1	0	
T-stage	T1/T2	158 (100%)	426 (99%)	0.54
	T3/T4	0	1 (1%)	
	Unknown	0	0	

*GE* denoted gene expression, *IDC* invasive ductal carcinoma, *ILC* invasive lobular carcinoma, *ER* oestrogen receptor, *HER2* ERBB2

Bold *p*-values denote *p*-values statitically significant at a 0.05 cut-off

## Discussion

In this study, we showed that patients of whom gene expression data were obtained had more often high-grade tumors and lymph node metastasis, features associated with a worse prognosis. It is important to acknowledge that, due to the selection of tumors with good quality samples and a high cellularity for gene expression studies, we select for a certain subgroup of tumors. Most likely, this selection bias is present in the majority of published gene expression studies for breast cancer.

Our findings are indeed in line with published literature. Cremoux et al. [13] studied (pre-) analytical steps in tissue handling by comparing different institutes. As in our study, they found that tumors suitable for gene expression profiling were more often high grade and of ductal subtype. Mook et al. observed a 17% dropout and concluded that the rejected samples were obtained from slightly smaller tumors [14]. Also Goetz et al. observed that women without expression data had more often a small tumor [15]. Together with the results of our validation dataset, there is substantial evidence towards the selection of larger and more aggressive tumors for gene expression studies.

In a systematic literature search that we performed prior to this study, finally resulting in 110 articles, 39% of the studies were indistinct about exclusion criteria and associated dropout rates, indicating unawareness. The remaining 61% did mention about exclusion criteria and numbers. These studies show a variety of dropout rates (1–83%, average 21%). Most gene expression profiles had been developed on frozen sample collections available at the biobank of the respective institute. These samples are a selection of relatively large and easily accessible tumors. Also, only samples that met the strict criteria of tumor RNA quality and quantity were used. Nowadays FFPE material from all laboratories (both biopsies and resection material), with very different protocols, can be measured by commercial available platforms, such as Mammaprint and OncotypeDx, and tumor percentage can be as low as 30% [16]. This is possible due to advances in the technique. However, we should be aware that such assays were originally not developed on samples with comparable characteristics, and validation on small samples with lower tumor cell percentage is warranted. In addition, also for research purposes, it is important to acknowledge that due to analytical requirements high-grade tumors might be overrepresented in GE datasets.

This study has some limitations. First, fresh frozen tumor samples were used. In general, it is more difficult to obtain fresh frozen material than FFPE tumor material, resulting in a higher dropout rate. Second, this study was done on pre-treatment biopsies, which yield smaller quantities of tumor material than resection specimens. Third, this study was performed in the neoadjuvant setting, which results in the selection of locally advanced tumors. Consequently, we could not look at stage I or stage IV tumors. Strong points of our study are that samples were obtained from a consecutive cohort of neoadjuvant treated patients, and not on a highly selected clinical trial population. Furthermore, we had an independent cohort for validation purposes that corroborated our findings. Although there were some differences in the way the samples were collected (resections versus biopsies, mailed transport versus snap frozen), the validation cohort consisted of early stage breast cancer samples and had information on grade, enabling us to validate our main finding in an independent cohort. Finally, all samples were from one institute and handled by one dedicated technician to preclude variability in centre or in lab handling.

In conclusion, we showed that breast cancers for which gene expression data were successfully obtained were associated with a higher grade and with lymph node metastasis, due to the selection of samples with a high tumor percentage and good quality RNA. These tumors have, on average, a more aggressive phenotype and a relatively poor prognosis. In general, when interpreting test results, it is important to realize that patient populations for which GE profiles are used, often differ substantially from the ones in which they were originally developed, particularly when using a development cohort consisting of frozen tumor tissue and a test cohort consisting of FFPE samples. At this point, it is uncertain what the impact might be on treatment decisions in the clinic.

## Electronic supplementary material

Below is the link to the electronic supplementary material.

Supplementary material 1 (PDF 397 kb)
