# Supplementary material for: Enrichment of high-grade tumors in breast cancer gene expression studies
Source: Breast Cancer Res Treat. 2017 Dec 18;168(2):327–35. doi: 10.1007/s10549-017-4622-9 (PMC5838139; doi:10.1007/s10549-017-4622-9)

Supplementary Tables and Figures

Enrichment of high grade tumors in breast cancer gene expression studies

Breast Cancer Research and Treatment

van Seijen M[a,b,1], Mooyaart AL[a,c,1], Mulder L[a], Hoogstraat ML[a,e], Drukker CA[f], Loo CE[g], Pouw B[f], Sonke GS[h], Wesseling J[a,d,2], Lips EH[a,d,2]*

*Corresponding author: E.H.Lips, The Netherlands Cancer Institute, Plesmanlaan 121, 1066 CX Amsterdam, The Netherlands, e.lips@nki.nl, Tel: +31 (0)20 5122045, Fax: +31 (0)20 5122050.

a) *Department of Molecular Pathology, the Netherlands Cancer Institute, Plesmanlaan 121, 1066 CX Amsterdam, the Netherlands,*
b) *Department of Pathology, VU University Medical Centre, De Boelelaan 1117, 1081 HV Amsterdam, the Netherlands*
c) *Department of Pathology, Erasmus University Medical Center, Wytemaweg 80 3015 CN Rotterdam, the Netherlands,*
d) *Department of Pathology, the Netherlands Cancer Institute, Plesmanlaan 121, 1066 CX Amsterdam, the Netherlands*
e) *Department of Molecular Carcinogenesis, the Netherlands Cancer Institute, Plesmanlaan 121, 1066 CX Amsterdam, the Netherlands*
f) *Department of Surgery, the Netherlands Cancer Institute, Plesmanlaan 121, 1066 CX Amsterdam, the Netherlands*
g) *Department of Radiology, the Netherlands Cancer Institute, Plesmanlaan 121, 1066 CX Amsterdam, the Netherlands*
h) *Department of Medical Oncology, the Netherlands Cancer Institute, Plesmanlaan 121, 1066 CX Amsterdam, the Netherlands*

[1] *Shared first authors*

[2] *Shared senior authors*

Supplementary Table 1 Characteristics of patients with gene expression profiles versus patients without gene expression profiles separated by subtype

GE denoted gene expression, IDC invasive ductal carcinoma, ILC invasive lobular carcinoma, ER estrogen receptor, HER2 ERBB2, T-stage tumor stadium, N-stage N stadium, SUV standardized uptake value

**Luminal**		n=364<br>no GE data available<br>(n=182)	GE data available<br>(n=182)	p-value
Age	<50 yr	95 (53%)	104 (57%)	0.44
	>=50 yr	84 (47%)	78 (43%)	
	Unknown	3	0	
Histology	IDC	135 (80%)	139 (82%)	0.74
	ILC	33 (20%)	31 (18%)	
	Unknown	14	12	
Grade	Grade 1 & 2	122 (88%)	95 (65%)	**<0.001**
	Grade 3	16 (12%)	52 (35 %)	
	Unknown	44	35	
T-stage	T1/T2	120 (67%)	120 (66%)	0.88
	T3/T4	60 (33%)	62(34%)	
	Unknown	2	0	
N-stage	neg	44 (24%)	37 (20%)	0.35
	pos	136 (76%)	145 (80%)	
	Unknown	2	0	
Response (breast)	NR or PR	143 (79%)	147 (81%)	0.75
	(n)pCR	37 (21 %)	35 (19%)	
	Unknown	2	0	
Response (breast and lymph node)	No pCR	168 (93 %)	174 (96%)	0.34
	pCR	12 (7%)	8 (4%)	
	Unknown	2	0	
Ki-67	>15%	94 (67%)	93 (68%)	0.76
	≤15%	47 (33%)	43 (32%)	
	Unknown	41	46	
Mass (MRI)	Non-mass	29 (60%)	27 (68%)	0.49
	Mass	19 (40%)	13 (33%)	
	Unknown	134	142	
Maximal SUV uptake mean (sd)		6.12 (2.99)	7.85 (4.17)	**0.03**
Unknown		133	142	

**HER2**		no GE data available (n=73)	GE data available (n=96)	p-value
		n=169		
Age	<50 yr	37 (51%)	54 (57%)	0.43
	>=50 yr	36 (49%)	41 (43%)	
	Unknown	0	1	
Histology	IDC	45 (94%)	68 (97%)	0.37
	ILC	3 (6%)	2 (3%)	
	Unknown	25	26	
Grade	Grade 1 & 2	34 (60%)	40 (44%)	0.07
	Grade 3	23 (40%)	50 (56%)	
	Unknown	16	6	
T-stage	T1/T2	45 (62%)	65 (69%)	0.31
	T3/T4	28 (38%)	29 (31%)	
	Unknown	0	2	
N-stage	neg	18 (25%)	17 (18%)	0.30
	pos	55 (75%)	77 (82%)	
	Unknown	0	2	
Response (breast)	NR or PR	22 (31%)	28 (30%)	0.92
	(n)pCR	50 (69%)	66 (70%)	
	Unknown	1	2	
Response (breast and lymph node)	No pCR	41 (56%)	53 (56%)	0.96
	pCR	32 (44%)	42 (44%)	
	Unknown	0	1	
Ki-67	>15%	7 (25%)	5 (21%)	0.72
	≤15%	21 (75%)	19 (79%)	
	Unknown	45	72	
Mass (MRI)	Non-mass	13 (68%)	14 (74%)	0.72
	Mass	6 (32%)	5 (26%)	
	Unknown	54	77	
Maximal SUV uptake mean (sd)		5·75 (2·80)	7·1 (2·52)	0.12
Unknown		53	76	

Triple negative		n=204<br>no GE data available (n=91)	GE data available (n=113)	p-value
Age	<50 yr	56 (63%)	79 (70%)	0.30
	>=50 yr	33 (37%)	34 (30%)	
	Unknown	2	0	
Histology	IDC	64 (97%)	84 (99%)	0.42
	ILC	2 (3%)	1 (1%)	
	Unknown	25	28	
Grade	Grade 1 & 2	28 (41%)	23 (25%)	**0.04**
	Grade 3	41 (59%)	68 (75%)	
	Unknown	22	22	
T-stage	T1/T2	67 (76%)	85 (76%)	0.97
	T3/T4	21 (24%)	27 (24%)	
	Unknown	3	1	
N-stage	neg	41 (46%)	40 (36%)	0.14
	pos	48 (54%)	72 (64%)	
	Unknown	2	1	
Response (breast)	NR or PR	37 (42%)	47 (42%)	0.95
	(n)pCR	51 (58%)	66 (58%)	
	Unknown	3	0	
Response (breast and lymph node)	No pCR	61 (69%)	66 (58%)	0.14
	pCR	28 (32%)	47 (42%)	
	Unknown	2	0	
Ki-67	>15%	2 (5%)	0 (0%)	0.15
	≤15%	39 (95%)	41 (100%)	
	Unknown	50	72	
Mass (MRI)	Non-mass	4 (22%)	12 (32%)	0.43
	Mass	14 (78%)	25 (68%)	
	Unknown	73	76	
Maximal SUV uptake mean (sd)		11.71 (6.63)	13.97 (8.65)	0.33
Unknown		73	76	

Supplementary Table 2 Multivariate cox proportional hazard analysis of the risk of recurrence (recurrence free survival) for clinical markers and a succesful gene expression profile.

GE denoted gene expression, IDC invasive ductal carcinoma, ILC invasive lobular carcinoma, ER estrogen receptor, HER2 ERBB2, T-stage tumor stadium, N-stage N stadium

Variable		event/total n	HR (95% CI)	p-value
	<50 year	72/426	1.00	0.15
Age2	>= 50 year	62/306	1.33 (0.90-1.96)	
	Neg	58/269	1.00	**0.03**
ER	Pos	77/463	0.62 (0.40-0.94)	
	Neg	112/561	1.00	**0.001**
HER2	Pos	23/169	0.41 (0.24-0.69)	
	Grade 1 or 2	55/341	1.00	0.23
Grade2	Grade 3	56/250	1.30 (0.85-1.96)	
	T1/T2	85/502	1.00	0.22
T_stage2	T3/T4	48/227	1.28 (0.86-1.90)	
	Neg	19/198	1.00	**<0.001**
N_stage	Pos	115/533	3.10 (1.76-5.48)	
	No GE data available	55/343	1.00	0.33
GEdata	GE data available	80/391	1.23 (0.82-1.85)	

Supplementary Fig. 1 Flow diagram of included patients in validation dataset (RASTER study).

DCIS denoted ductal carcinoma in situ

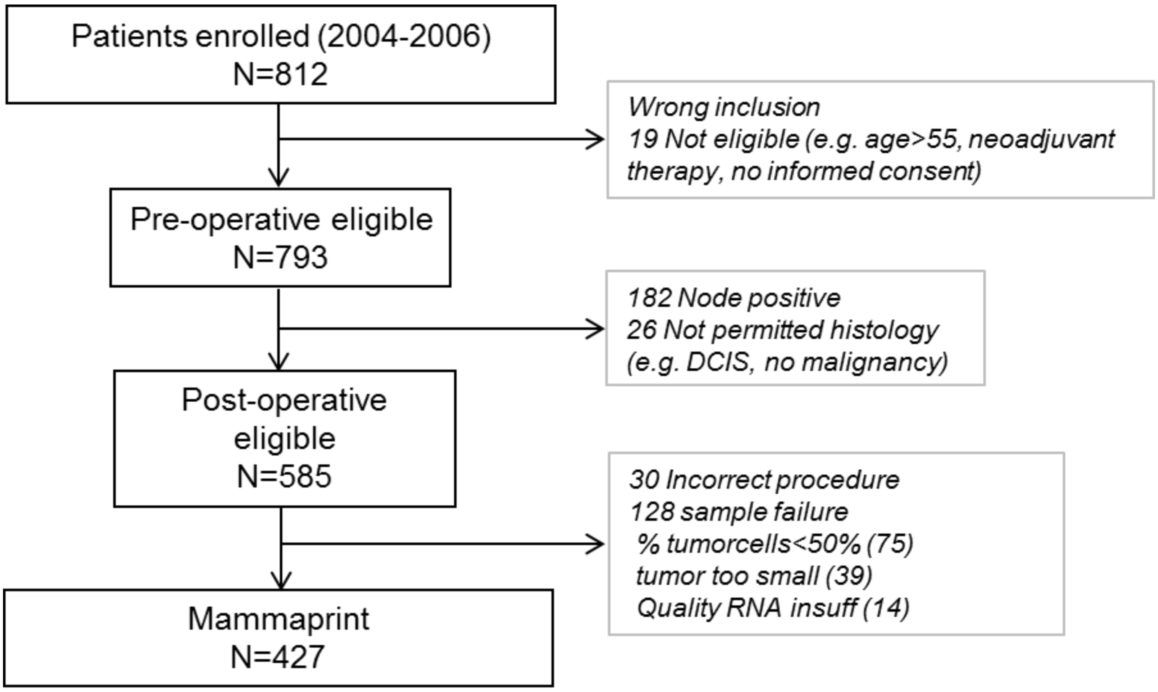

Supplement: Supplementary file 1 — Supplementary material 1 (PDF 397 kb) [file 10549_2017_4622_MOESM1_ESM.pdf]
